# Algorithm-based assessment of T-cell dysfunction and exclusion to forecast ICB sensitivity in pediatric brain ependymoma

**DOI:** 10.1007/s11060-025-05384-4

**Published:** 2025-12-19

**Authors:** Matteo Palermo, Luca Massimi, Giampiero Tamburrini, Alessandro Olivi, Francesco Doglietto, Alessio Albanese, Carmelo Lucio Sturiale

**Affiliations:** 1 Department of Neurosurgery, Fondazione Policlinico Universitario A. Gemelli IRCCS, Università Cattolica del Sacro Cuore, 00168 Rome, Italy; 2 Department of Pediatric Neurosurgery, Fondazione Policlinico A. Gemelli IRCCS, Università Cattolica del Sacro Cuore, 00168 Roma, Italy

**Keywords:** Ependymoma, Immune-checkpoint-blocker, TIDE, Immunotherapy

## Abstract

**Background:**

Pediatric brain ependymomas are brain tumors difficult to cure despite the advancements in surgery and radiotherapy. Immunotherapies, specifically immune checkpoint blockades (ICB), are traditionally recognized to have a limited efficacy against cold microenvironment as the ones of ependymomas. By employing the Tumor Immune Dysfunction and Exclusion (TIDE) scoring system, this study tries to predict the responsiveness to ICB across molecular subgroups, recurrent/primary presentation and hot vs. cold subtypes.

**Methods:**

Four GEO datasets from NCBI public library were selected for this study. In total, 150 RNA-bulk sequences of pediatric ependymomas were analyzed (PF-A = 125, ZFTA-RELA = 23, YAP1 = 2). The TIDE algorithm was applied to quantify cytotoxic T-cell infiltration, dysfunction, and exclusion, estimating ICB response probabilities. Group differences were calculated with Kruskal–Wallis and Fisher’s exact tests (*p* < 0.05).

**Results:**

60% of ependymomas were predicted ICB responders. ZFTA-RELA tumors showed significantly lower TIDE scores than PF-A (–0.099 ± 0.263 vs. 0.060 ± 0.316; *p* = 0.008) and a higher response rate (78.3% vs. 56.0%; *p* = 0.063). RELA-fusion tumors exhibited reduced T-cell dysfunction (–0.235 ± 0.221 vs. − 0.098 ± 0.152; *p* < 0.001). Recurrent tumors demonstrated lower TIDE scores (–0.070 ± 0.328 vs. 0.081 ± 0.296; *p* < 0.001) and greater predicted response (75.6% vs. 52.4%; *p* = 0.011). Responders overall had lower TIDE, dysfunction, and exclusion values (all *p* < 0.01).

**Conclusions:**

Pediatric ependymomas are not uniformly immune-silent. ZFTA-RELA and recurrent tumors exhibit a “primed but suppressed” immune phenotype, where the immune machinery is present but functionally restrained, suggesting greater susceptibility to ICB, whereas PF-A tumors remain immune-excluded and may require microenvironmental modulation to achieve immunotherapy benefit.

**Supplementary Information:**

The online version contains supplementary material available at 10.1007/s11060-025-05384-4.

## Introduction

Ependymomas can be classified in distinct molecular subgroups based on genomic and epigenomic profiling [1, 2]. In pediatric patients, the key subtypes include posterior fossa ependymoma Group A (PF-A) and Group B (PF-B), as well as supratentorial ependymomas with RELA fusion or YAP1 fusion [1, 3, 4]. PF-A ependymomas are typical of young children, with an aggressive course and poor survival (50% 5y-OS), whereas PF-B tumors, seen more often in older children/adolescents, show a milder tumorigenicity and a more favorable prognosis [4]. Similarly, roughly 70% of pediatric supratentorial ependymomas harbor C11orf95-RELA fusions and tend to be clinically aggressive, while those with YAP1-MAMLD1 fusions, common in infants, show excellent outcomes [4]. Yet, curative outcomes are possible in many cases; however, certain high-risk molecular subsets, like PF-A, remain largely refractory to durable cure even with maximal surgery and radiation. This concern underlines the need for a subgroup-stratified analysis to address differences in response to therapies [2, 4].

Over the past decade, cancer immunotherapy has revolutionized oncology, with immune checkpoint blockers (ICBs) producing long-lasting remissions in several malignancies [5]. However, only a minority of patients respond to checkpoint blockade, and their clinical responses are exclusive to highly immunogenic tumors with many neoantigens [6]. Tumor immune evasion (TCE) occurs primarily through T-cell dysfunction (TCD) or through exclusion of T cells from the tumor microenvironment [6].

The Tumor Immune Dysfunction and Exclusion (TIDE) algorithm uses transcriptomic data to model these two mechanisms: inducing T-cell dysfunction in T cell-inflamed tumors and preventing T-cell infiltration in non-inflamed tumors, to predict a tumor’s likelihood response to immunotherapies [7].

Application of ICB therapy to pediatric brain tumors such as ependymoma has so far been very limited [6]. Ependymomas typically exhibit low mutation loads and an immunologically cold tumor microenvironment with minimal T-cell infiltration [8]. While no ICB is broadly FDA-approved for pediatric brain tumors, these agents may still be used in selected hypermutated gliomas (including ependymomas) where off-label administration has been reported. Nonetheless, emerging research suggests that immune milieu may vary across ependymoma subgroups thus possibly suggesting different therapeutic responses [6]. However, evidence is still scarce, and mechanisms associated with immunological response versus resistance in these tumors are still poorly understood.

As a result, this study aims to evaluate potential therapeutic differences in subgroup-stratified analysis of the host immune microenvironment, thereby providing preliminary evidence for possible future clinical trials.

## Methods

We searched the NCBI GEO database for bulk RNA‑seq datasets containing pediatric ependymoma samples, using the query filters “rnaseq counts” and “ependymoma.” By the last update on October 16, 2025, this search yielded 12 candidate datasets. Two independent reviewers (CLS and MP) screened these datasets separately. Following a collegial re-evaluation, they reached an agreement on those to be included; any disagreements were resolved by a third reviewer (AO) following a separate reassessment of the databases. Because our analysis focused on patient tumor transcriptomes, we excluded datasets that included cell cultures (4), xenografts (1), or animal models (1). One additional dataset was excluded because its raw and processed data were not available. A standard method comparing submitted Sequence Read Archive (SRA) files to the human genome (GCA_000001405.15) produced TPM-normalized RNA-seq counts for all included samples. Samples with less than 50% alignment were excluded. The limma package in R was used to compensate for batch effects (Combat) in the TPM count matrices (Table 1- Supplementary Material). One-hundred-and-fifty tissue samples from 4 GEO databases were selected after screening (GSE241396, GSE243682, GSE181162, and GSE156619).

**Table 1:** Comparison of clinical characteristics and TIDE-derived immune metrics across ependymoma molecular subgroups

	PF-A (*n* = 125)	ZFTA-RELA (*n* = 23)	Overall (*n* = 148)		*p*-value (CI)
**TIDE**			**0.008 (0.020–0.297)**		
Mean (SD)	0.060 (0.316)	-0.099 (0.263)	0.035 (0.313)		
Range	1.850	1.310	1.850		
**ICB Response**			0.063 (0.929–10.302)		
Non-Responder	55 (44.0%)	5 (21.7%)	60 (40.5%)		
Responder	70 (56.0%)	18 (78.3%)	88 (59.5%)		
**Dysfunction score**			**< 0.001 (0.063–0.210)**		
Mean (SD)	-0.098 (0.152)	-0.235 (0.221)	-0.119 (0.171)		
Range	0.820	0.910	0.980		
**Presentation**			0.147 (0.703–5.384)		
Primary	90 (72.0%)	13 (56.5%)	103 (69.6%)		
Recurrent	35 (28.0%)	10 (43.5%)	45 (30.4%)		
**Exclusion score**			0.426 (-0.045–0.128)		
Mean (SD)	0.533 (0.189)	0.491 (0.218)	0.526 (0.193)		
Range	0.980	0.800	1.010		
**Immunological Status**			0.252 (-0.380–1.772)		
Cold	57 (45.6%)	7 (30.4%)	54 (43.2%)		
Hot	68 (54.4%)	16 (69.6%)	84 (56.8%)		

Then, data were analyzed using the TIDE algorithm. TIDE, developed by Jiang et al. (2018), is a transcriptome-based predictive model for ICB response that has been shown to outperform traditional biomarkers and tumor mutational burden. This model predicts response based on two main mechanisms: T cell exclusion (TCE) and T cell dysfunction (TCD). TCE defines failure of immune cells to infiltrate cold tumors, whereas TCD describes a state in which tumor-infiltrating lymphocytes lose their cytotoxic ability despite their hot milieu. The algorithm estimates cytotoxic CD8⁺ T-cell activity by calculating the mean expression of core CTL-associated genes (CD8A, CD8B, GZMA, GZMB, PRF1). Tumors are classified as immunologically *hot* when at least two out of three criteria exceed cohort-based thresholds, defined as IFNG expression above the cohort mean, CTL abundance above the mean, and MDSC levels below the mean. Tumors are predicted to respond to ICB if they are immunologically hot with no evidence of TCD, or immunologically cold with no evidence of TCE. This refers exclusively to immune checkpoint blockade and does not extend to other immunotherapeutic modalities such as vaccines, oncolytic viruses, or cell-based therapies. Because the TIDE output scores are relative and not calibrated to absolute biological thresholds, CTL infiltration values were dichotomized based on the cohort mean, classifying samples with CD8 expression above the mean as high (Hot) and those below as low (Cold); this dataset-specific normalization ensures comparability across samples within the same analysis [7]. By correlating each tumor’s expression profile with established gene signatures of exhausted T cells and immunosuppressive cell types, the algorithm computes TCD and TCE scores for each sample.

TIDE uses reference datasets from lung cancer and melanoma patients treated with anti-PD-1 or anti-CTLA-4 therapies to classify tumors as likely responders or non-responders. In particular, response was defined per RECIST criteria as stable disease or partial/complete tumor reduction, while progressive disease was considered non-response. We implemented TIDE using the TIDEpy Python package (Jiang et al.) with the default threshold settings [7]. Statistical analyses were performed in JASP v19.3.0: p-values below 0.05 were considered significant, using Kruskal–Wallis tests for continuous variables and Fisher’s exact tests for categorical variables.

## Results

### Baseline characteristics

A total of four datasets were included in the analysis, including 150 human pediatric ependymoma tissue samples with available RNA-seq count data. All samples were intracranial. Spinal ependymomas were excluded because they constitute a biologically distinct disease entity with different genomic drivers, immune microenvironmental features, and clinical behavior compared to intracranial pediatric ependymomas, thereby warranting separate dedicated investigation. Information on mean patient age was not retrievable due to missing demographic data across the datasets. Sex distribution was reported in three of the four datasets, totaling 33 females and 97 males. Among the samples, 2 harbored YAP1 mutations, 125 were classified as PF-A, and 23 exhibited ZFTA-RELA fusions. Only GSE243682 reported PF-A subtype 1 (24 samples) vs. subtype 2 (17) samples. No tumors belonging to the PF-B subtype were identified. Additionally, 104 samples corresponded to primary tumors, while 46 represented recurrent cases. However, the YAP1 group (*n* = 2) was excluded from the univariate analyses due to its insufficient sample size; data from these samples were only reported descriptively (Table 2 Supplementary Material).

**Table 2:** Comparison of clinical characteristics and TIDE-derived immune metrics across PF-A1 vs. PF-A2 tumors

	PF-A1 (*n* = 24)	PF-A2 (*n* = 17)	Overall (*n* = 41)		*p*-value (CI)
**TIDE**			**0.161 (0.028–0.320)**		
Mean (SD)	0.131 (0.287)	0.065 (0.291)	0.103 (0.287)		
Range	0.990	0.940	1.020		
**ICB Response**			0.208 (-0.507–2.432)		
Non-Responder	14 (58.3%)	6 (35.3%)	20 (48.7%)		
Responder	10 (41.2%)	11 (64.7%)	21 (51.2%)		
**Dysfunction score**			0.106 (0.028–0.293)		
Mean (SD)	-0.041 (0.102)	-0.077 (0.084)	-0.056 (0.095)		
Range	0.440	0.320	0.440		
**Presentation**			0.629 (-4.846–1.819)		
Primary	21 (87.5%)	16 (94.7%)	37 (90.2%)		
Recurrent	3 (12.5%)	1 (5.8%)	4 (9.7%)		
**Exclusion score**			0.624 (0.051–0.144)		
Mean (SD)	0.534 (0.123)	0.514 (0.047)	0.526 (0.155)		
Range	0.480	0.770	0.770		
**Immunological Status**			0.745 (-1.146–1.803)		
Cold	16 (66.7%)	10 (58.8%)	26 (63.4%)		
Hot	8 (33.3%)	7 (41.2%)	15 (36.6%)		

### Molecular subgroups’ differences

Across the two molecular subgroups with available data, mean TIDE scores and dysfunctional scores differed between groups (Table 1).

**Fig. 1:**
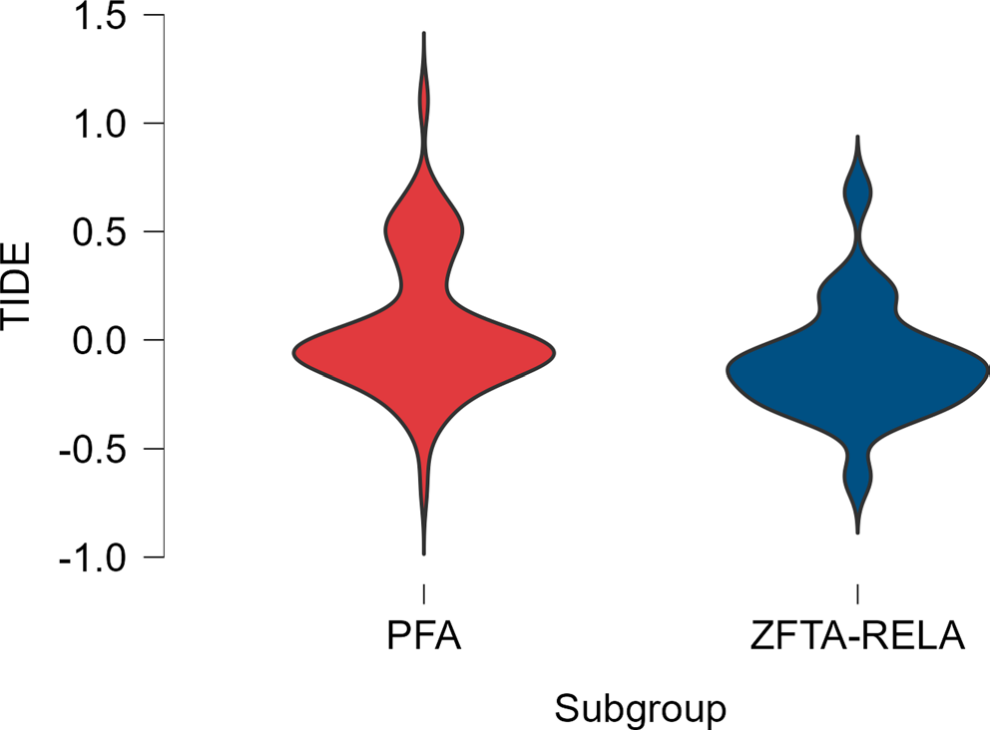
Distribution of TIDE scores based on molecular subtypes (*p* = 0.008)

Notably, PF-A tumors reported a mean TIDE score of 0.060 ± 0.316, whereas mean ZFTA-RELA tumors’ score was − 0.099 ± 0.263 (*p* = 0.008) (Fig. 1).

Predicted ICB response rates were similar between subtypes: in the PF-A group, 55 cases (44.0%) were classified as non-responders and 70 as responders (56.0%), compared to 5 (21.7%) non-responders and 18 (78.3%) responders in the ZFTA-RELA group. However, this distribution of ICB response status showed a trend towards significance between PF-A and ZFTA-RELA groups (*p* = 0.063). Similarly, tumor presentation (primary vs. recurrent) and exclusion scores did not differ significantly between subgroups (*p* = 0.147 and *p* = 0.426).

A statistically significant difference was found in the TCD scores (*p* < 0.001). The mean dysfunction score was − 0.098 ± 0.152 in PF-A tumors versus − 0.235 ± 0.221 in ZFTA-RELA tumors, with ranges of 0.820 and 0.910, respectively. Finally, the immunological status did not differ significantly between PF-A and ZFTA-RELA fusion ependymomas, with most ZFTA-RELA tumors classified as cold, while 54.4% of PF-A tumors were also cold. (*p* = 0.252).

Instead, no statistical significance was found when analyzing the subset of datasets distinguishing between PF-A1 and PF-A2 in all domains (Table 2).

### Primary versus recurrent tumors

When grouped by tumor presentation, ependymomas showed significant differences in TIDE scores, predicted immunogenic response, and dysfunction score distribution.

**Fig. 2:**
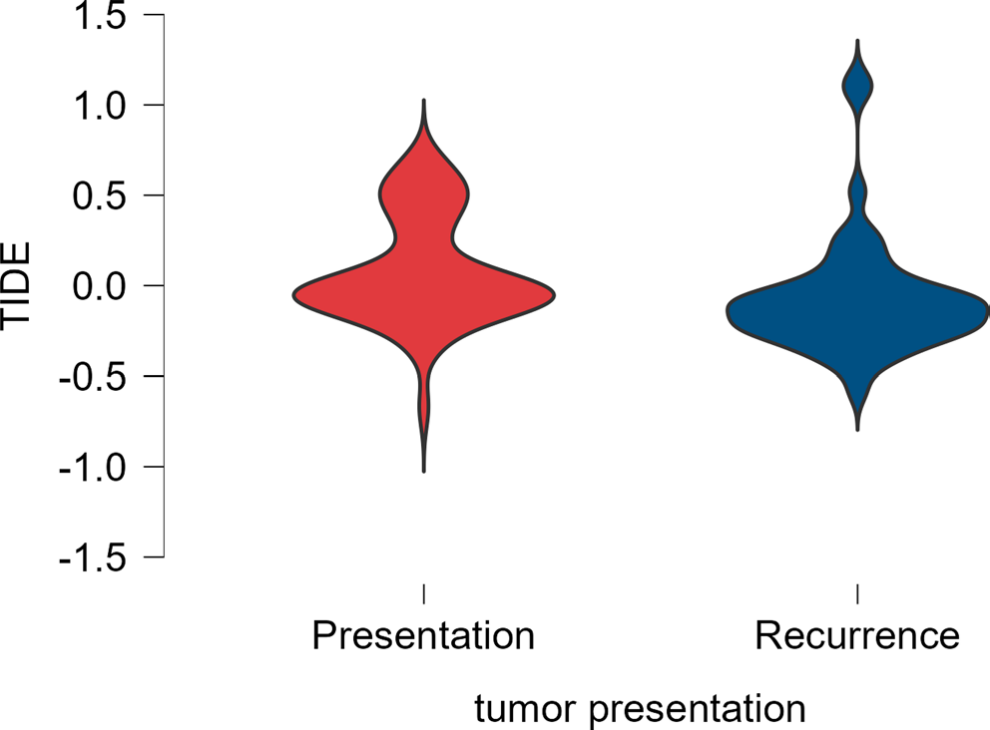
Distribution of TIDE scores based on tumor presentation (primary vs. recurrent) (*P* < 0.001)

Specifically, the mean TIDE score was significantly different between primary (0.081 ± 0.296) versus recurrent tumors (-0.070 ± 0.328), with an overall mean of 0.035 ± 0.313 (*p* < 0.001) (Table 3; Fig. 2).

**Table 3:** Comparison of clinical characteristics and TIDE-derived immune metrics across primary vs. recurrent tumors

	Primary (*n* = 103)	Recurrent (*n* = 45)	Overall (*n* = 148)	*p*-value (CI)
**TIDE**			**< 0.001 (0.043–0.259)**	
Mean (SD)	0.081 (0.296)	-0.070 (0.328)	0.035 (0.313)	
Range	1.420	1.720	1.850	
**ICB Response**			**0.011 (0.196–1.915)**	
Non-Responder	49 (47.6%)	11 (24.4%)	60 (40.5%)	
Responder	54 (52.4%)	34 (75.6%)	88 (59.5%)	
**Dysfunction score**			**< 0.001 (0053–0.168)**	
Mean (SD)	-0.086 (0.156)	-0.196 (0.181)	-0.119 (0.171)	
Range	0.810	0.980	0.980	
**Subgroup Analysis**			0.147 (-0.353–1.684)	
PF-A	90 (87.4%)	35 (77.8%)	125 (84.5%)	
ZFTA-RELA	13 (12.6%)	10 (22.2%)	23 (15.5%)	
**Exclusion score**			0.335 (-0155–0.021)	
Mean (SD)	0.512 (0.162)	0.559 (0.249)	0.526 (0.193)	
Range	0.770	1.010	1.010	
**Immunological Status**			**< 0.001 (0.643–2.472)**	
Cold	55 (53.4%)	9 (20.0%)	64 (43.2%)	
Hot	48 (46.6%)	36 (80.0%)	84 (56.8%)	

Predicted ICB response differed by presentation: there were 49 (47.6%) non-responders and 54 (52.4%) responders in the primary tumor group, versus 11 (24.4%) non-responders and 34 (75.6%) responders in the recurrent tumor group. This distribution of ICB response status showed a significant difference between primary and recurrent ependymomas (*p* = 0.011).

The mean dysfunction score analysis varied significantly between groups, with primary tumors (-0.086 ± 0.156) scoring higher than recurrent tumors (-0.196 ± 0.181) (*p* < 0.001). Differently, molecular subtype compositionand exclusion scores were similar between primary and recurrent groups (*p* = 0.147,and *p* = 0.335). Differently, most recurrent tumors were defined as hot compared to primary tumors (80.0% vs. 46.6%) (*p* < 0.001).

### ICB responders versus non-responders

When grouped by predicted response to ICB, ependymomas showed significant differences in all tested domains (Table 4). The mean TIDE score was higher in predicted non-responders (0.321 ± 0.275) than in ICB responders (− 0.160 ± 0.138), with an overall mean of 0.035 ± 0.313 (*p* < 0.001).

**Table 4:** Comparison of clinical characteristics and TIDE-derived immune metrics across responders vs. non-responders

	Non-Responder (*n* = 60)	ICB Responder (*n* = 88)	Overall (*n* = 148)	*p*-value (CI)
**TIDE**			**< 0.001 (0.414–0.549)**	
Mean (SD)	0.321 (0.275)	-0.160 (0.138)	0.035 (0.313)	
Range	1.130	0.710	1.850	
**Subtype Analysis**			**0.063 (-0.074–2.332)**	
PF-A	55 (91.7%)	70 (94.8%)	125 (84.5%)	
ZFTA-RELA	5 (8.3%)	18 (20.45%)	23 (15.5%)	
**Dysfunction score**			**< 0.001 (0.047–0.156)**	
Mean (SD)	-0.059 (0.196)	-0.160 (0.138)	-0.119 (0.171)	
Range	0.980	0.710	0.980	
**Presentation**			**0.011 (0.196–1.915)**	
Primary	49 (81.2%)	54 (61.4%)	103 (69.5%)	
Recurrent	11 (18.3%)	34 (38.6%)	45 (30.5%)	
**Exclusion score**			**0.004 (-0.143 - -0.018)**	
Mean (SD)	0.479 (0.181)	0.559 (0.195)	0.526 (0.193)	
Range	0.980	0.950	1.010	
**Immunological Status**			**0.007 (0.199–1.658)**	
Cold	34 (56.7%)	30 (34.1%)	64 (43.2%)	
Hot	26 (43.3%)	58 (65.9%)	84 (56.8%)	

Subtype analysis varied between groups, with most tumors in both non-responders (*n* = 55, 90.2%) and responders (*n* = 70, 78.7%) belonging to the PF-A subtype, while only a minority harbored the ZFTA-RELA fusion (8.2% in the non-responder group vs. 20.2% in the responder group; *p* = 0.063).

The dysfunction score showed a statistically significant difference between responders (-0.160 ± 0.138) and non-responders (-0.059 ± 0.196) (*p* < 0.001). Tumor presentation also differed significantly by response group, as 49 (81.2%) non-responding tumors were primary and 11 (18.3%) recurrent, compared to 54 (61.4%) primary and 34 (38.6%) recurrent tumors among responders (*p* = 0.011). The exclusion score was significantly higher in responders (0.559 ± 0.195) than in non-responders (0.479 ± 0.181; *p* = 0.004).

Importantly, most cold tumors were not responsive to ICB (56.7%), while most hot tumors were predicted otherwise (65.9%) (*p* = 0.007).

### Cold versus hot tumors

When grouped by immunological status, both the TIDE score and the dysfunction score differed significantly (*p* = 0.004 and *p* = 0.001, respectively) (Table 5). Specifically, the mean TIDE score among cold tumors was − 0.117 ± 0.299, whereas hot tumors showed a mean of -0.028 ± 0.310. Similarly, the mean dysfunction score was lower in cold tumors (–0.067 ± 0.111) compared to hot tumors (–0.159 ± 0.197). Similarly, significant differences were also noted in ICB response (*p* = 0.006); primary/recurrent tumor analysis (*p* < 0.001) and exclusion scores (*p* < 0.001).

**Table 5:** Comparison of clinical characteristics and TIDE-derived immune metrics across hot vs. cold tumors

	Cold (*n* = 64)	Hot (*n* = 84)	Overall (*n* = 148)	*p*-value (CI)
**TIDE**			**0.004 (0.044–0.245)**	
Mean (SD)	0.117 (0.299)	-0.028 (0.310)	0.035 (0.313)	
Range	-0.400	-0.710	1.850	
**ICB Response**			**0.006 (0.199–1.658)**	
Non-Responder	34 (53.1%)	26 (30.9%)	60 (40.5%)	
Responder	30 (46.9%)	58 (69.1%)	88 (59.5%)	
**Exclusion score**			**< 0.001 (-0.202 - -0.084)**	
Mean (SD)	0.445 (0.135)	0.588 (0.208)	0.526 (0.193)	
Range	0.560	0.930	1.010	
**Presentation**			**< 0.001 (0.643–2.472)**	
Primary	55 (85.9%)	48 (57.1%)	103 (69.6%)	
Recurrent	9 (14.1%)	36 (42.99%)	45 (30.4%)	
**Dysfunction score**			**0.001 (0.038–0.146)**	
Mean (SD)	-0.067 (0.111)	-0.159 (0.197)	-0.119 (0.171)	
Range	0.460	0.980	0.980	

## Discussion

Ependymomas are rare gliomas that predominantly affect children, constituting only about 4–5% of all primary central nervous system tumors [9]. They rank as the third most common pediatric brain malignancy [10], arising in locations such as the supratentorial region or posterior fossa. Despite aggressive surgery and radiotherapy, only 50–70% of ependymoma patients achieve durable cure [10]. Tumors that cannot be fully resected at diagnosis tend to have poor prognosis: most of these children suffer relapses and eventually die of refractory disease. As a consequence, immunotherapy, particularly ICB, has gained interest as a new avenue for treating pediatric brain tumors [10].

### PF-A versus RELA-fusion ependymoma

Recent profiling studies reveal *subgroup-specific* variations in the immune landscapes of ependymomas [11]. Our analysis of 148 pediatric ependymomas revealed significant heterogeneity in immune microenvironment and predicted response to ICB across molecular subgroups. This is remarkable given that ependymomas have traditionally been viewed as immunologically cold tumors with sparse cytotoxic T-cell infiltration [12].

Interestingly, our study revealed that the projected response rate for supratentorial ependymomas with the ZFTA-RELA fusion was substantially higher than that of PF-A ependymomas. Compared to 56.0% of PF-A ependymomas, over 78.3% of RELA-fusion ependymomas were projected ICB responders. This implies that, in contrast to the PFA subgroup, the tumors in the RELA subgroup exhibit an ICB-sensitive phenotype. Nevertheless, while our model suggests that ZFTA-RELA and recurrent tumors may theoretically respond to ICB, several biological and clinical factors likely explain why clinical studies to date have not demonstrated therapeutic success. Our prediction is based on transcriptomic signatures rather than on functional evidence of response; therefore, even if the immune microenvironment appears favorable on a molecular level, multiple mechanisms may still prevent effective ICB activity. These include: (i) the highly immunosuppressive milieu of ependymoma, with myeloid-dominant infiltrates and scarce effector T-cells; (ii) low tumor mutational burden and limited neoantigenicity, reducing ICB target visibility; (iii) exhaustion or dysfunction of the small T-cell populations present; and (iv) limited drug penetration across the blood–brain and tumor–brain barriers.

Our results are consistent with growing evidence of a unique immune milieu in RELA-fusion ependymomas. In 2019, Witt et al. showed that PD-L1 expression was entirely absent in PF-A 10 and restricted to RELA-fusion ependymomas [10]. In addition, T-cells infiltrating RELA tumors also exhibited an exhausted phenotype [10]. These findings point to an immunosuppressed yet immunocompetent state in RELA-fusion tumors, implying that ependymomas may exist in a “*primed but suppressed*” condition, where the immune machinery is present but functionally restrained [6, 11].

This helps explain why even tumors that look cold under the microscope might still be capable of responding to immunotherapy once the right signals are restored. Consistent with this concept, our analysis showed that 64.0% of tumors classified as cold were predicted to respond to ICBs. In other words, some tumors that seem inactive based on immune cell infiltration may actually express genes linked to immune activation, such as interferon signaling, antigen presentation, or partially suppressed T-cell activity, that make them vulnerable to T-cell reactivation by ICB [13]. However, at the same time, factors like TGF-β, IL-10 and tumor-associated cells like macrophages and macroglia reduce their immunological capacity by restricting T-cell activation.

This idea of “*quiet but capable*” challenges the belief that all ependymomas are ICB-resistant, suggesting that some subtypes may have undiscovered weaknesses.

If these findings get validated, they could pave the way to future trials testing ICBs for specific molecular subgroups. However, at present, these observations remain purely speculative and require biological confirmation before informing therapeutic strategy. Additionally, validation would also suggest that specific metrics alone, such as T-cell counts, could underestimate the potential of immunotherapy for malignancy.

In sum, future research should explore how T-cell activity and interferon pathways reactivate the immune milieu of cold tumors in response to ICB.

### Primary vs. recurrent tumors and immune response

Primary vs. recurrent tumors analysis revealed a significant difference in ICB response prediction. The ICB response was more likely to occur in recurrent (75.6%) than in primary tumors (52.4%), suggesting that a more immune-responsive transcriptome profile is associated with tumor recurrence (*p* = 0.011). One possible explanation is that standard treatments, like surgery, radiation, chemotherapy, can alter the tumor biology in ways that increase immunogenicity. Recurrent tumors may accumulate a higher mutational burden and new neoantigens due to therapy-induced DNA damage [14]. This increase in neoantigens and tumor subclonal diversity could make recurrent tumors more recognizable to the immune system, thus more susceptible to checkpoint blockade.

Additionally, prior therapy might shift the tumor microenvironment: for example, radiation is known to upregulate MHC class I and increase inflammatory cytokines in tumors [12], potentially converting some cold tumors into “hotter” ones.

Our finding that recurrent ependymomas are more often ICB-responsive suggests that timing and treatment history matter for immunotherapy. It raises the question of whether ICB might be particularly effective as an adjuvant or salvage therapy after initial treatments, when the tumor may be primed by therapy-induced changes.

However, it is also possible that the aggressive biology of recurrent tumors, which have overcome initial therapy, could determine a more inflammatory microenvironment. As a result, enhanced RELA-fusion subtypes and other pro-immune factors may be responsible for the increased response rate seen in recurrent samples.

Either way, these differences in predicted response rates between primary and recurrent tumors further highlight the heterogeneity of ependymoma’s milieus.

Future clinical trials should take into account these variations in predicted response across subgroups and recurrent cases when testing a new immunotherapeutic for an ependymoma.

### Implications for future research and precision immunotherapy

The implications of this study for future research are vast. Bulk RNA sequencing and computational immune scoring (TIDE) has been proven effective in forecasting, as a supplementary tool, how well immunotherapy would work for ependymomas.

Moreover, our results confirm that a personalized immunotherapeutic approach is needed to account for the diversity of the ependymomas. This implies that future clinical trials should concentrate on the subgroups most likely to benefit from immunotherapy.

However, despite being intriguing, our preliminary results urge for experimental validation. Molecular studies will also help to clarify the pathways involved in reactivating or hyperactivating dormant T-cells in response to ICBs.

If successful, transcriptome-guided immunotherapy could transform the management of pediatric ependymomas, by offering more personalized and effective treatments.

### Limitations

This study has several limitations. First, the TIDE algorithm uses bulk RNA sequences for the analysis [7], thus lacks single-cell precision required to differentiate signals from various cellular pathways. Consequently, this approach was unable to discern variations among distinct tumor or immune cell populations [15]. We acknowledge that single-cell RNA-seq would represent the most accurate approach to assess T-cell exhaustion and immune exclusion in ependymoma. However, currently available scRNA-seq datasets are limited by small sample size, unbalanced molecular subgroup representation, lack of clinical metadata, and insufficient lymphocyte depth for reliable TIDE-based modeling. As such, scRNA-seq could not be integrated into immune-response prediction, and our analysis was necessarily performed using bulk RNA-seq cohorts, which remain the only sufficiently annotated data source available at this time.

Furthermore, the database search yielded no PF-B tumor samples. Although this limitation stems from dataset availability rather than study design, it may still introduce bias and should be considered when interpreting the generalizability of our conclusions. Additionally, we warrant for a cautious interpretation of the analysis distinguishing PF-A1 vs. PF-A2 tumors, due to limited sample sizes. Cautious interpretation of the results is warranted for the RELA subgroup due to the small sample size.

Information on prior treatment (surgery, radiation or combined) was not available in the collected datasets. This calls for cautious interpretation of our results.

Of note, TIDE was initially trained and validated using samples from non–small cell lung cancers and melanomas. Because it’s used beyond the context for which it was originally developed, its reliability in tumors different from the ones listed before remains questionable.

Moreover, TIDE only provides probabilistic estimates of the ICB response based on different gene expressions. However, this further limits the ability to discern between which pathways or cell types are driving these effects. Additionally, because transcriptomic analysis only captures a single snap of the tumor’s molecular profile, it fails to show how the interaction between the tumor and the immune system evolves over time.

Nevertheless, these results are purely theoretical and derive from a predictive model; therefore, prospective validation is necessary to confirm whether such response will be observed clinically.In sum, although the model represents a useful computational predictor, given its limitations these results warrant caution interpretation until validation.

## Conclusion

This study showed that ependymomas, despite being considered cold tumors, might respond differently to immunotherapies. ZFTA-RELA subtypes showed a potential stronger response to ICB compared to PF-A types. Additionally, recurrent tumors were also found to be more responsive compared to baseline. This suggests that a one-size-fit-all approach to tumors is not beneficial. Tailoring treatments based on tumor-specific characteristics, can help enhance precision and personalization in cancer care.

## Supplementary Information

Below is the link to the electronic supplementary material.

Supplementary Material 1: Batch correction (combat).

Supplementary Material 2: Summary of patient characteristics and data analysis.

## Data Availability

No datasets were generated or analysed during the current study.

## Abbreviations

CTL: Cytotoxic T lymphocyte
FDA: U.S. Food and Drug Administration
GEO: Gene Expression Omnibus
ICB: Immune Checkpoint Blockade
IRB: Institutional Review Board
JASP: Jeffreys’s Amazing Statistics Program
MHC: Major Histocompatibility Complex
NCBI: National Center for Biotechnology Information
OS: Overall survival
PD-1: Programmed Death-1
PD-L1: Programmed Death-Ligand 1
PF-A / PF-B: Posterior Fossa Group A / Group B ependymoma
RNA-seq: RNA sequencing; SRA: Sequence Read Archive
TCD: T-cell dysfunction
TCE: T-cell exclusion
TGF-β: Transforming Growth Factor Beta
TIDE: Tumor Immune Dysfunction and Exclusion
TPM: Transcripts per million
YAP1: Yes-associated protein 1
ZFTA-RELA: Zinc Finger Translocation Associated–RELA fusion
