# Supplementary figures and images for: Algorithm-based assessment of T-cell dysfunction and exclusion to forecast ICB sensitivity in pediatric brain ependymoma
Source: J Neurooncol. 2025 Dec 19;176(2):128. doi: 10.1007/s11060-025-05384-4 (PMC12717181; doi:10.1007/s11060-025-05384-4)

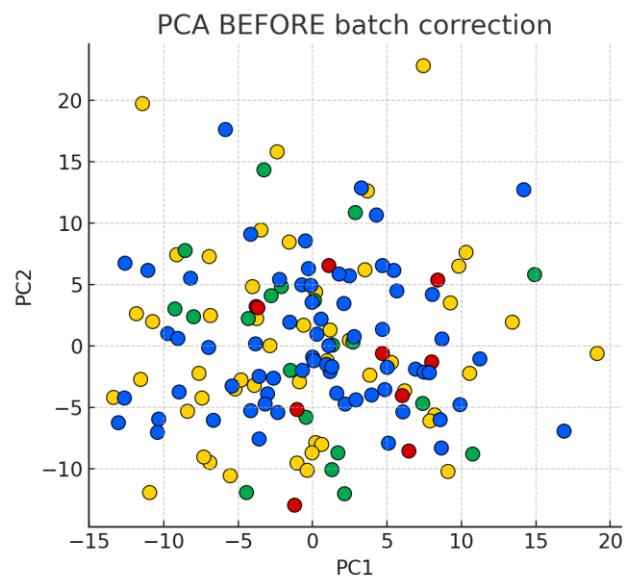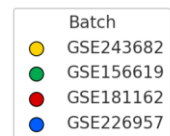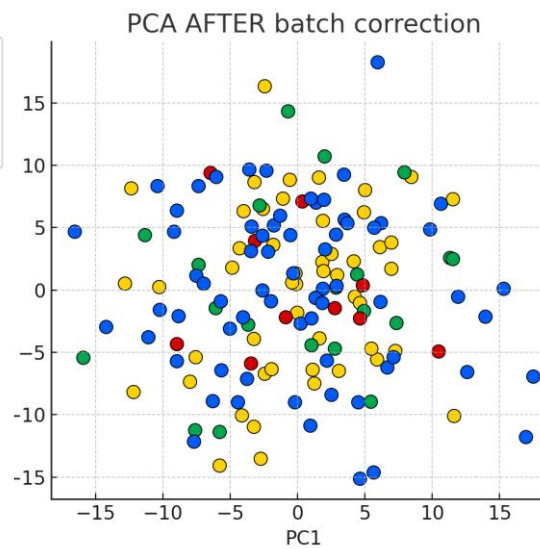

Supplement: Supplementary file 1 — Supplementary Material 1: Batch correction (combat). [file 11060_2025_5384_MOESM1_ESM.pdf]
