# Supplementary material for: Algorithm-based assessment of T-cell dysfunction and exclusion to forecast ICB sensitivity in pediatric brain ependymoma
Source: J Neurooncol. 2025 Dec 19;176(2):128. doi: 10.1007/s11060-025-05384-4 (PMC12717181; doi:10.1007/s11060-025-05384-4)
Supplement: Supplementary file 2 — Supplementary Material 2: Summary of patient characteristics and data analysis. [file 11060_2025_5384_MOESM2_ESM.docx]

**Table 2 - Supplementary Material:** Summary of patient characteristics and data analysi

| **Patient** | **Subgroup** | **ST/PF** | **Sex** | **presentation/recurrence** | **No benefits** | **Responder** | **TIDE** | **IFNG** | **MSI Expr Sig** | **Merck18** | **CD274** | **CD8** | **TYPE** | **CTL.flag** | **Dysfunction** | **Exclusion** | **MDSC** | **CAF** | **TAM M2** |
| --- | --- | --- | --- | --- | --- | --- | --- | --- | --- | --- | --- | --- | --- | --- | --- | --- | --- | --- | --- |
| GSM7794059 | PF-A | PF | M | Presentation | FALSE | TRUE | -0.1 | 22.48 | 1 | 14.85 | 0.24 | 0.22 | HOT | TRUE | -0.1 | 0.75 | 0.04 | 0.08 | -0.01 |
| GSM7794066 | PF-A | PF | M | Presentation | FALSE | TRUE | -0.08 | 14.77 | 1 | 10.13 | 0.31 | 0.31 | HOT | TRUE | -0.08 | 0.54 | 0.04 | 0.05 | 0 |
| GSM7794074 | PF-A | PF | M | Recurrence | FALSE | TRUE | -0.26 | 7.29 | 1 | 7.46 | 0.07 | 0.53 | COLD | TRUE | -0.26 | 0.72 | 0.05 | 0.05 | 0.02 |
| GSM7794084 | ZFTA-RELA | ST | M | Presentation | FALSE | TRUE | -0.05 | 23.45 | 1 | 15.04 | 2.41 | 0.1 | HOT | TRUE | -0.05 | 0.57 | 0.03 | 0.06 | 0 |
| GSM7794109 | ZFTA-RELA | ST | F | Presentation | FALSE | FALSE | 0.04 | 13.92 | 1 | 7.42 | 0.33 | 0.13 | COLD | TRUE | 0.04 | 0.6 | 0.04 | 0.05 | 0.01 |
| GSM7794119 | ZFTA-RELA | ST | M | Recurrence | FALSE | TRUE | -0.09 | 10.74 | 1 | 9.46 | 0.43 | 0.15 | HOT | TRUE | -0.09 | 0.61 | 0.05 | 0.05 | 0.01 |
| GSM7794127 | ZFTA-RELA | ST | M | Presentation | FALSE | FALSE | 0.68 | 10.36 | 1 | 5.81 | 0.36 | 0 | HOT | FALSE | -0.02 | 0.68 | 0.04 | 0.06 | 0.01 |
| GSM7794133 | PF-A | PF | M | Presentation | FALSE | FALSE | 0.65 | 6.38 | 1 | 7.95 | 0.03 | 0.35 | HOT | FALSE | -0.06 | 0.65 | 0.03 | 0.06 | 0.02 |
| GSM7794135 | PF-A | PF | M | Presentation | FALSE | TRUE | -0.05 | 55.39 | 1 | 25.64 | 4.62 | 0.06 | COLD | TRUE | -0.05 | 0.63 | 0.04 | 0.07 | -0.02 |
| GSM7794142 | PF-A | PF | F | Presentation | FALSE | FALSE | 0.53 | 30.78 | 1 | 12.65 | 0.26 | 0.19 | HOT | FALSE | 0.05 | 0.53 | 0.02 | 0.06 | 0 |
| GSM7794146 | ZFTA-RELA | ST | M | Presentation | FALSE | TRUE | -0.05 | 5.63 | 1 | 6.86 | 0.26 | 0.14 | HOT | TRUE | -0.05 | 0.55 | 0.04 | 0.05 | 0.01 |
| GSM7794148 | PF-A | PF | F | Presentation | FALSE | TRUE | -0.28 | 10.98 | 1 | 10.81 | 0.23 | 0.45 | HOT | TRUE | -0.28 | 0.6 | 0.06 | 0.04 | 0.01 |
| GSM7794152 | PF-A | PF | M | Presentation | FALSE | TRUE | -0.01 | 78.39 | 1 | 25.67 | 1.06 | 0.42 | HOT | TRUE | -0.01 | 0.7 | 0.04 | 0.07 | -0.01 |
| GSM7794153 | PF-A | PF | M | Presentation | FALSE | TRUE | -0.34 | 11.83 | 1 | 7.5 | 0.27 | 0.47 | COLD | TRUE | -0.34 | 0.64 | 0.05 | 0.05 | 0.02 |
| GSM7794174 | ZFTA-RELA | ST | M | Presentation | FALSE | TRUE | -0.13 | 13.35 | 1 | 9.23 | 0.67 | 0.04 | HOT | TRUE | -0.13 | 0.73 | 0.04 | 0.05 | 0.01 |
| GSM7794175 | PF-A | PF | F | Presentation | FALSE | TRUE | -0.14 | 60.15 | 1 | 13.88 | 0.54 | 0.17 | COLD | TRUE | -0.14 | 0.95 | 0.05 | 0.1 | -0.01 |
| GSM7794192 | PF-A | PF | M | Presentation | FALSE | FALSE | 0.53 | 12.16 | 1 | 7.79 | 0.17 | 0.55 | COLD | FALSE | 0.02 | 0.53 | 0.02 | 0.06 | 0 |
| GSM7794213 | PF-A | PF | F | Presentation | FALSE | FALSE | 0.02 | 86.23 | 1 | 35.08 | 3.07 | 0.28 | HOT | TRUE | 0.02 | 0.6 | 0.03 | 0.08 | -0.02 |
| GSM7794061 | PF-A | PF | M | Recurrence | FALSE | TRUE | -0.11 | 16.67 | 1 | 8.57 | 0.16 | 0.67 | HOT | TRUE | -0.11 | 0.67 | 0.04 | 0.05 | 0.01 |
| GSM7794062 | PF-A | PF | M | Recurrence | FALSE | TRUE | -0.02 | 32.73 | 1 | 13.42 | 0.43 | 1.1 | COLD | TRUE | -0.02 | 0.27 | 0.02 | 0.02 | 0 |
| GSM7794072 | PF-A | PF | M | Presentation | FALSE | FALSE | 0.31 | 15.78 | 1 | 15.83 | 0.01 | 0.68 | COLD | FALSE | 0.03 | 0.31 | 0.02 | 0.03 | 0.01 |
| GSM7794100 | PF-A | PF | F | Presentation | FALSE | FALSE | 0.38 | 12.8 | 1 | 10.7 | 0.15 | 0.69 | HOT | FALSE | 0 | 0.38 | 0.02 | 0.04 | 0.01 |
| GSM7794103 | ZFTA-RELA | ST | F | Presentation | FALSE | TRUE | -0.14 | 33.66 | 1 | 17.14 | 0.24 | 1.22 | COLD | TRUE | -0.14 | 0.86 | 0.06 | 0.06 | 0.02 |
| GSM7794112 | ZFTA-RELA | ST | M | Presentation | FALSE | TRUE | 0 | 49.03 | 1 | 21.86 | 0.6 | 1.2 | COLD | TRUE | 0 | 0.73 | 0.04 | 0.08 | 0 |
| GSM7794114 | PF-A | PF | M | Recurrence | FALSE | TRUE | -0.07 | 120.01 | 1 | 27.71 | 1.14 | 1.24 | HOT | TRUE | -0.07 | 0.67 | 0.03 | 0.08 | -0.01 |
| GSM7794131 | PF-A | PF | M | Presentation | FALSE | FALSE | 0.56 | 6.61 | 1 | 7.8 | 0.06 | 3.03 | HOT | FALSE | -0.01 | 0.56 | 0.03 | 0.05 | 0.01 |
| GSM7794143 | PF-A | PF | F | Presentation | FALSE | TRUE | -0.03 | 93 | 1 | 22.82 | 1.37 | 0.52 | COLD | TRUE | -0.03 | 0.49 | 0.02 | 0.06 | -0.01 |
| GSM7794144 | PF-A | PF | F | Presentation | FALSE | FALSE | 0.46 | 14.42 | 1 | 9.07 | 0.18 | 0.2 | COLD | FALSE | 0.06 | 0.46 | 0.01 | 0.05 | 0 |
| GSM7794147 | PF-A | PF | F | Presentation | FALSE | TRUE | -0.09 | 15.17 | 1 | 13.29 | 0.12 | 0.59 | COLD | TRUE | -0.09 | 0.58 | 0.03 | 0.05 | 0.02 |
| GSM7794149 | PF-A | PF | M | Presentation | FALSE | FALSE | 0.48 | 12.65 | 1 | 10.56 | 0.45 | 0.59 | COLD | FALSE | 0.06 | 0.48 | 0.01 | 0.06 | 0 |
| GSM7794150 | PF-A | PF | M | Presentation | FALSE | FALSE | 0.41 | 13.22 | 1 | 7.36 | 0.13 | 1.33 | HOT | FALSE | -0.08 | 0.41 | 0.02 | 0.04 | 0.01 |
| GSM7794159 | PF-A | PF | M | Presentation | FALSE | FALSE | 0.01 | 73.15 | 1 | 28.2 | 0.26 | 0.74 | COLD | TRUE | 0.01 | 0.51 | 0.04 | 0.06 | -0.03 |
| GSM7794163 | PF-A | PF | M | Presentation | FALSE | FALSE | 0.68 | 35.8 | 1 | 17.8 | 0.18 | 0.59 | HOT | FALSE | -0.1 | 0.68 | 0.03 | 0.07 | 0 |
| GSM7794164 | PF-A | PF | M | Presentation | FALSE | TRUE | -0.06 | 15.8 | 1 | 7.82 | 0.22 | 0.59 | COLD | TRUE | -0.06 | 0.44 | 0.02 | 0.04 | 0.01 |
| GSM7794165 | PF-A | PF | F | Presentation | FALSE | TRUE | -0.08 | 33.55 | 1 | 17.19 | 0.2 | 0.29 | HOT | TRUE | -0.08 | 0.48 | 0.03 | 0.04 | 0.01 |
| GSM7794167 | PF-A | PF | M | Presentation | FALSE | FALSE | 0.1 | 94.29 | 1 | 46.75 | 0.87 | 1.04 | COLD | TRUE | 0.1 | 0.33 | 0.01 | 0.05 | -0.03 |
| GSM7794168 | PF-A | PF | M | Presentation | FALSE | FALSE | 0.01 | 17.52 | 1 | 10.93 | 0.12 | 0.9 | COLD | TRUE | 0.01 | 0.55 | 0.03 | 0.05 | 0.01 |
| GSM7794170 | PF-A | PF | M | Presentation | FALSE | TRUE | -0.08 | 29.59 | 1 | 12.92 | 0.4 | 0.98 | COLD | TRUE | -0.08 | 0.27 | 0.01 | 0.03 | 0.01 |
| GSM7794171 | PF-A | PF | M | Presentation | FALSE | TRUE | -0.16 | 26.59 | 1 | 12.89 | 0.34 | 0.43 | COLD | TRUE | -0.16 | 0.48 | 0.04 | 0.04 | 0 |
| GSM7794172 | PF-A | PF | M | Presentation | FALSE | FALSE | 0.5 | 7.98 | 1 | 7.65 | 0.13 | 0.16 | COLD | FALSE | 0.02 | 0.5 | 0.03 | 0.05 | 0 |
| GSM7794177 | PF-A | PF | M | Presentation | FALSE | TRUE | -0.18 | 18.78 | 1 | 9.91 | 0.11 | 0.99 | COLD | TRUE | -0.18 | 0.6 | 0.03 | 0.05 | 0.01 |
| GSM7794179 | ZFTA-RELA | ST | M | Presentation | FALSE | TRUE | -0.1 | 54.45 | 1 | 20.96 | 2.37 | 0.31 | HOT | TRUE | -0.1 | 0.32 | 0.02 | 0.03 | 0 |
| GSM7794181 | PF-A | PF | M | Presentation | FALSE | FALSE | 0.03 | 38.2 | 1 | 14.5 | 0.57 | 0.3 | HOT | TRUE | 0.03 | 0.4 | 0.02 | 0.04 | 0 |
| GSM7794182 | PF-A | PF | M | Presentation | FALSE | TRUE | -0.09 | 26.67 | 1 | 19.97 | 0.21 | 0.6 | HOT | TRUE | -0.09 | 0.69 | 0.03 | 0.06 | 0.01 |
| GSM7794184 | PF-A | PF | M | Presentation | FALSE | FALSE | 0.06 | 28.23 | 1 | 11.08 | 0.24 | 0.17 | HOT | TRUE | 0.06 | 0.46 | 0.01 | 0.05 | 0 |
| GSM7794187 | PF-A | PF | M | Presentation | FALSE | FALSE | 0.03 | 7 | 1 | 9.63 | 0.03 | 0.87 | COLD | TRUE | 0.03 | 0.3 | 0.02 | 0.02 | 0.01 |
| GSM7794188 | PF-A | PF | M | Presentation | FALSE | TRUE | -0.04 | 11.32 | 1 | 12.05 | 0.06 | 0.99 | COLD | TRUE | -0.04 | 0.18 | 0.01 | 0.01 | 0.02 |
| GSM7794196 | PF-A | PF | M | Presentation | FALSE | FALSE | 0.34 | 16.88 | 1 | 10.95 | 1.23 | 0.38 | HOT | FALSE | -0.12 | 0.34 | 0.02 | 0.04 | -0.01 |
| GSM7794211 | PF-A | PF | M | Presentation | FALSE | TRUE | -0.14 | 12.01 | 1 | 6.67 | 0.24 | 0.59 | HOT | TRUE | -0.14 | 0.66 | 0.03 | 0.06 | 0.02 |
| GSM7794212 | PF-A | PF | M | Presentation | FALSE | FALSE | 0.56 | 10.48 | 1 | 9.06 | 0.13 | 1.66 | COLD | FALSE | -0.01 | 0.56 | 0.03 | 0.05 | 0.01 |
| GSM4735032 | PF-A | PF | NR | Recurrence | FALSE | TRUE | -0.23 | 17.81 | 1 | 13.65 | 0.69 | 0.33 | COLD | TRUE | -0.23 | 0.82 | 0.06 | 0.07 | 0 |
| GSM4735033 | ZFTA-RELA | ST | NR | Recurrence | FALSE | TRUE | -0.33 | 14.33 | 1 | 8.94 | 0.89 | 0.02 | COLD | TRUE | -0.33 | 0.62 | 0.04 | 0.05 | 0 |
| GSM4735038 | ZFTA-RELA | ST | NR | Recurrence | FALSE | TRUE | -0.25 | 11.68 | 1 | 11.14 | 0.4 | 0.05 | HOT | TRUE | -0.25 | 0.93 | 0.05 | 0.09 | 0.01 |
| GSM4735042 | ZFTA-RELA | ST | NR | Recurrence | FALSE | TRUE | -0.36 | 19.95 | 1 | 11.2 | 0.77 | 0.07 | COLD | TRUE | -0.36 | 0.57 | 0.04 | 0.05 | 0 |
| GSM4735024 | PF-A | PF | NR | Recurrence | FALSE | FALSE | 0.16 | 5.16 | 1 | 6.14 | 0.45 | 0.53 | HOT | FALSE | -0.12 | 0.16 | 0.01 | 0.02 | 0 |
| GSM4735025 | PF-A | PF | NR | Recurrence | FALSE | TRUE | -0.39 | 19.93 | 1 | 8.57 | 0.3 | 0.28 | COLD | TRUE | -0.39 | 0.33 | 0.03 | 0.03 | 0.01 |
| GSM4735026 | PF-A | PF | NR | Recurrence | FALSE | TRUE | -0.1 | 112.33 | 1 | 34.3 | 1.33 | 0.87 | COLD | TRUE | -0.1 | 0.51 | 0.02 | 0.06 | -0.01 |
| GSM4735027 | PF-A | PF | NR | Recurrence | FALSE | FALSE | 1.07 | 12.52 | 1 | 8.7 | 1.25 | 1.01 | HOT | FALSE | -0.44 | 1.07 | 0.07 | 0.08 | 0.02 |
| GSM4735028 | PF-A | PF | NR | Presentation | FALSE | TRUE | -0.09 | 29.55 | 1 | 16.73 | 0.56 | 0.23 | COLD | TRUE | -0.09 | 0.2 | 0.01 | 0.03 | -0.01 |
| GSM4735029 | PF-A | PF | NR | Presentation | FALSE | TRUE | -0.3 | 26.99 | 1 | 17.53 | 0.44 | 0.3 | COLD | TRUE | -0.3 | 0.45 | 0.03 | 0.04 | -0.01 |
| GSM4735030 | PF-A | PF | NR | Recurrence | FALSE | TRUE | -0.4 | 20.36 | 1 | 11.57 | 0.34 | 0.36 | COLD | TRUE | -0.4 | 0.33 | 0.03 | 0.02 | 0.02 |
| GSM4735031 | PF-A | PF | NR | Recurrence | FALSE | TRUE | -0.21 | 40.23 | 1 | 19.4 | 0.55 | 1.05 | HOT | TRUE | -0.21 | 0.42 | 0.03 | 0.04 | 0 |
| GSM4735034 | ZFTA-RELA | ST | NR | Recurrence | FALSE | TRUE | -0.21 | 17.16 | 1 | 9.72 | 0.66 | 0.24 | HOT | TRUE | -0.21 | 0.13 | 0.01 | 0.01 | 0.02 |
| GSM4735035 | ZFTA-RELA | ST | NR | Recurrence | FALSE | TRUE | -0.28 | 32.76 | 1 | 14.84 | 0.77 | 0.12 | HOT | TRUE | -0.28 | 0.36 | 0.02 | 0.03 | 0.01 |
| GSM4735036 | ZFTA-RELA | ST | NR | Recurrence | FALSE | TRUE | -0.29 | 19.56 | 1 | 12.25 | 0.6 | 0.23 | HOT | TRUE | -0.29 | 0.5 | 0.03 | 0.05 | 0.01 |
| GSM4735037 | ZFTA-RELA | ST | NR | Presentation | FALSE | TRUE | -0.14 | 10.5 | 1 | 11.82 | 0.56 | 0.09 | HOT | TRUE | -0.14 | 0.44 | 0.03 | 0.04 | 0 |
| GSM4735039 | ZFTA-RELA | ST | NR | Recurrence | FALSE | TRUE | -0.12 | 39.3 | 1 | 22.98 | 1.17 | 0.47 | HOT | TRUE | -0.12 | 0.48 | 0.02 | 0.06 | -0.01 |
| GSM4735040 | ZFTA-RELA | ST | NR | Presentation | FALSE | TRUE | -0.3 | 10.53 | 1 | 8.46 | 1.42 | 0.15 | HOT | TRUE | -0.3 | 0.38 | 0.03 | 0.03 | 0.01 |
| GSM4735041 | ZFTA-RELA | ST | NR | Recurrence | FALSE | FALSE | 0.19 | 16.96 | 1 | 8.16 | 0.36 | 0.27 | HOT | FALSE | -0.26 | 0.19 | 0.02 | 0.01 | 0.02 |
| GSM4735043 | ZFTA-RELA | ST | NR | Presentation | FALSE | TRUE | -0.2 | 6.01 | 1 | 4.99 | 0.31 | 0.03 | HOT | TRUE | -0.2 | 0.35 | 0.02 | 0.03 | 0.01 |
| GSM5491992 | ZFTA-RELA | ST | M | Recurrence | FALSE | FALSE | 0.27 | 49.77 | 1 | 19.47 | 3.16 | 0 | HOT | FALSE | -0.87 | 0.27 | 0.06 | 0.08 | 0.01 |
| GSM5491993 | ZFTA-RELA | ST | M | Presentation | FALSE | TRUE | -0.63 | 32.88 | 1 | 12.24 | 2.95 | 0.23 | HOT | TRUE | -0.63 | 0.22 | 0.04 | 0.07 | 0.01 |
| GSM5491994 | ZFTA-RELA | ST | F | Presentation | FALSE | FALSE | 0.21 | 29.99 | 1 | 10.97 | 0.76 | 0.02 | HOT | FALSE | -0.62 | 0.21 | 0.05 | 0.06 | 0.01 |
| GSM5491995 | YAP1 | ST | F | Recurrence | FALSE | FALSE | 0.43 | 55.98 | 1 | 16.95 | 3.12 | 0.12 | HOT | FALSE | -0.9 | 0.43 | 0.05 | 0.08 | 0.02 |
| GSM5491996 | YAP1 | ST | F | Presentation | FALSE | TRUE | -0.44 | 20.81 | 1 | 12.84 | 2.99 | 1.9 | HOT | TRUE | -0.44 | 0.36 | 0.05 | 0.08 | 0.01 |
| GSM5491997 | PF-A | PF | M | Recurrence | FALSE | TRUE | -0.58 | 41.02 | 1 | 14.47 | 0.53 | 0.34 | COLD | TRUE | -0.58 | 0.21 | 0.04 | 0.06 | 0.01 |
| GSM5491998 | PF-A | PF | F | Presentation | FALSE | FALSE | 0.22 | 52.76 | 1 | 17.63 | 0.41 | 0.5 | COLD | FALSE | -0.3 | 0.22 | 0.03 | 0.06 | 0.01 |
| GSM5491999 | PF-A | PF | F | Presentation | FALSE | FALSE | 0.27 | 31.44 | 1 | 12.53 | 0.18 | 0.48 | HOT | FALSE | -0.7 | 0.27 | 0.07 | 0.06 | 0.01 |
| GSM5492000 | PF-A | PF | F | Presentation | FALSE | FALSE | 0.26 | 51 | 1 | 23.4 | 0.9 | 0.61 | COLD | FALSE | -0.48 | 0.26 | 0.03 | 0.07 | 0.01 |
| GSM5492001 | PF-A | PF | M | Presentation | FALSE | TRUE | -0.71 | 18.84 | 1 | 7.18 | 0 | 1.2 | HOT | TRUE | -0.71 | 0.3 | 0.05 | 0.03 | 0.01 |
| GSM7088806 | PF-A | PF | M | Presentation | FALSE | TRUE | -0.1 | 22.48 | 1 | 14.85 | 0.24 | 0.22 | HOT | TRUE | -0.1 | 0.75 | 0.04 | 0.08 | -0.01 |
| GSM7088807 | PF-A | PF | M | Presentation | FALSE | TRUE | -0.08 | 14.77 | 1 | 10.13 | 0.31 | 0.31 | HOT | TRUE | -0.08 | 0.54 | 0.04 | 0.05 | 0 |
| GSM7088810 | PF-A | PF | M | Recurrence | FALSE | TRUE | -0.26 | 7.29 | 1 | 7.46 | 0.07 | 0.53 | HOT | TRUE | -0.26 | 0.72 | 0.05 | 0.05 | 0.02 |
| GSM7088812 | PF-A | PF | M | Presentation | FALSE | FALSE | 0.02 | 86.23 | 1 | 35.08 | 3.07 | 0.28 | HOT | TRUE | 0.02 | 0.6 | 0.03 | 0.08 | -0.02 |
| GSM7088813 | PF-A | PF | M | Presentation | FALSE | FALSE | 0.53 | 12.16 | 1 | 7.79 | 0.17 | 0.55 | HOT | FALSE | 0.02 | 0.53 | 0.02 | 0.06 | 0 |
| GSM7088819 | PF-A | PF | M | Presentation | FALSE | FALSE | 0.65 | 6.38 | 1 | 7.95 | 0.03 | 0.35 | HOT | FALSE | -0.06 | 0.65 | 0.03 | 0.06 | 0.02 |
| GSM7088820 | PF-A | PF | M | Presentation | FALSE | TRUE | -0.05 | 55.39 | 1 | 25.64 | 4.62 | 0.06 | HOT | TRUE | -0.05 | 0.63 | 0.04 | 0.07 | -0.02 |
| GSM7088822 | PF-A | PF | F | Presentation | FALSE | FALSE | 0.53 | 30.78 | 1 | 12.65 | 0.26 | 0.19 | HOT | FALSE | 0.05 | 0.53 | 0.02 | 0.06 | 0 |
| GSM7088828 | PF-A | PF | F | Presentation | FALSE | TRUE | -0.28 | 10.98 | 1 | 10.81 | 0.23 | 0.45 | HOT | TRUE | -0.28 | 0.6 | 0.06 | 0.04 | 0.01 |
| GSM7088831 | PF-A | PF | F | Presentation | FALSE | TRUE | -0.09 | 27.16 | 1 | 12.52 | 0.16 | 0.51 | HOT | TRUE | -0.09 | 0.8 | 0.05 | 0.07 | 0.01 |
| GSM7088834 | PF-A | PF | M | Presentation | FALSE | TRUE | -0.01 | 78.39 | 1 | 25.67 | 1.06 | 0.42 | HOT | TRUE | -0.01 | 0.7 | 0.04 | 0.07 | -0.01 |
| GSM7088835 | PF-A | PF | M | Presentation | FALSE | TRUE | -0.34 | 11.83 | 1 | 7.5 | 0.27 | 0.47 | HOT | TRUE | -0.34 | 0.64 | 0.05 | 0.05 | 0.02 |
| GSM7088855 | PF-A | PF | M | Recurrence | FALSE | TRUE | -0.31 | 10.12 | 1 | 7.44 | 0.21 | 0.38 | HOT | TRUE | -0.31 | 0.65 | 0.04 | 0.05 | 0.02 |
| GSM7088857 | PF-A | PF | M | Presentation | FALSE | TRUE | -0.03 | 20 | 1 | 10.9 | 0.54 | 0.52 | HOT | TRUE | -0.03 | 0.71 | 0.04 | 0.07 | 0.01 |
| GSM7088863 | PF-A | PF | M | Presentation | FALSE | TRUE | -0.05 | 30.53 | 1 | 14.59 | 0.13 | 0.4 | COLD | TRUE | -0.05 | 0.71 | 0.04 | 0.07 | 0 |
| GSM7088868 | PF-A | PF | M | Recurrence | FALSE | FALSE | 0.01 | 93.41 | 1 | 29.03 | 0.23 | 0.28 | COLD | TRUE | 0.01 | 0.7 | 0.04 | 0.08 | -0.01 |
| GSM7088874 | PF-A | PF | F | Presentation | FALSE | TRUE | -0.14 | 60.15 | 1 | 13.88 | 0.54 | 0.17 | COLD | TRUE | -0.14 | 0.95 | 0.05 | 0.1 | -0.01 |
| GSM7088877 | PF-A | PF | M | Recurrence | FALSE | TRUE | -0.18 | 13.15 | 1 | 8.48 | 0.08 | 0.22 | COLD | TRUE | -0.18 | 0.86 | 0.06 | 0.05 | 0.02 |
| GSM7088878 | PF-A | PF | M | Recurrence | FALSE | TRUE | -0.16 | 26.98 | 1 | 8.57 | 0.12 | 0.14 | COLD | TRUE | -0.16 | 1.08 | 0.07 | 0.08 | 0.01 |
| GSM7088881 | PF-A | PF | M | Recurrence | FALSE | TRUE | -0.19 | 131.86 | 1 | 31.13 | 0.71 | 0.33 | HOT | TRUE | -0.19 | 0.85 | 0.05 | 0.08 | 0 |
| GSM7088882 | PF-A | PF | M | Recurrence | FALSE | TRUE | -0.21 | 68.21 | 1 | 23.66 | 0.69 | 0.18 | HOT | TRUE | -0.21 | 0.77 | 0.05 | 0.08 | -0.01 |
| GSM7088883 | PF-A | PF | M | Recurrence | FALSE | FALSE | 1.14 | 11.8 | 1 | 10.98 | 0.38 | 0.2 | COLD | FALSE | -0.28 | 1.14 | 0.06 | 0.1 | 0.01 |
| GSM7088885 | PF-A | PF | M | Recurrence | FALSE | TRUE | -0.11 | 28.33 | 1 | 21.14 | 0.23 | 0.51 | COLD | TRUE | -0.11 | 0.71 | 0.04 | 0.06 | 0.01 |
| GSM7088805 | PF-A | PF | M | Presentation | FALSE | TRUE | -0.08 | 17.32 | 1 | 15.28 | 0.4 | 0.71 | HOT | TRUE | -0.08 | 0.38 | 0.02 | 0.04 | 0 |
| GSM7088808 | PF-A | PF | M | Presentation | FALSE | FALSE | 0.03 | 9.12 | 1 | 12.92 | 0.23 | 0.52 | HOT | TRUE | 0.03 | 0.43 | 0.02 | 0.05 | 0 |
| GSM7088809 | PF-A | PF | M | Presentation | FALSE | FALSE | 0.56 | 10.48 | 1 | 9.06 | 0.13 | 1.66 | COLD | FALSE | -0.01 | 0.56 | 0.03 | 0.05 | 0.01 |
| GSM7088811 | PF-A | PF | M | Presentation | FALSE | FALSE | 0.31 | 15.78 | 1 | 15.83 | 0.01 | 0.68 | HOT | FALSE | 0.03 | 0.31 | 0.02 | 0.03 | 0.01 |
| GSM7088814 | PF-A | PF | F | Presentation | FALSE | FALSE | 0.38 | 12.8 | 1 | 10.7 | 0.15 | 0.69 | COLD | FALSE | 0 | 0.38 | 0.02 | 0.04 | 0.01 |
| GSM7088816 | PF-A | PF | M | Presentation | FALSE | FALSE | 0.71 | 7.84 | 1 | 8.2 | 0.2 | 0.74 | COLD | FALSE | -0.06 | 0.71 | 0.04 | 0.06 | 0.02 |
| GSM7088817 | PF-A | PF | F | Recurrence | FALSE | TRUE | -0.07 | 120.01 | 1 | 27.71 | 1.14 | 1.24 | COLD | TRUE | -0.07 | 0.67 | 0.03 | 0.08 | -0.01 |
| GSM7088818 | PF-A | PF | M | Presentation | FALSE | FALSE | 0.56 | 6.61 | 1 | 7.8 | 0.06 | 3.03 | HOT | FALSE | -0.01 | 0.56 | 0.03 | 0.05 | 0.01 |
| GSM7088821 | PF-A | PF | M | Presentation | FALSE | TRUE | -0.03 | 10.37 | 1 | 6.07 | 0.13 | 0.23 | COLD | TRUE | -0.03 | 0.39 | 0.02 | 0.04 | 0 |
| GSM7088823 | PF-A | PF | F | Presentation | FALSE | TRUE | -0.03 | 93 | 1 | 22.82 | 1.37 | 0.52 | HOT | TRUE | -0.03 | 0.49 | 0.02 | 0.06 | -0.01 |
| GSM7088824 | PF-A | PF | F | Recurrence | FALSE | TRUE | -0.07 | 48.34 | 1 | 16.36 | 0.7 | 1.48 | HOT | TRUE | -0.07 | 0.33 | 0.02 | 0.04 | 0 |
| GSM7088825 | PF-A | PF | F | Presentation | FALSE | FALSE | 0.46 | 14.42 | 1 | 9.07 | 0.18 | 0.2 | HOT | FALSE | 0.06 | 0.46 | 0.01 | 0.05 | 0 |
| GSM7088826 | PF-A | PF | F | Presentation | FALSE | FALSE | 0.02 | 50.35 | 1 | 16.7 | 0.77 | 0.73 | HOT | TRUE | 0.02 | 0.61 | 0.02 | 0.07 | 0 |
| GSM7088827 | PF-A | PF | F | Presentation | FALSE | TRUE | -0.09 | 15.17 | 1 | 13.29 | 0.12 | 0.59 | HOT | TRUE | -0.09 | 0.58 | 0.03 | 0.05 | 0.02 |
| GSM7088829 | PF-A | PF | F | Recurrence | FALSE | TRUE | -0.19 | 5.3 | 1 | 7.65 | 0.07 | 0.66 | COLD | TRUE | -0.19 | 0.46 | 0.03 | 0.03 | 0.02 |
| GSM7088830 | PF-A | PF | M | Presentation | FALSE | FALSE | 0.48 | 12.65 | 1 | 10.56 | 0.45 | 0.59 | HOT | FALSE | 0.06 | 0.48 | 0.01 | 0.06 | 0 |
| GSM7088832 | PF-A | PF | F | Recurrence | FALSE | TRUE | -0.02 | 82.11 | 1 | 28.75 | 0.48 | 1.55 | HOT | TRUE | -0.02 | 0.68 | 0.03 | 0.08 | 0 |
| GSM7088833 | PF-A | PF | M | Presentation | FALSE | FALSE | 0.41 | 13.22 | 1 | 7.36 | 0.13 | 1.33 | HOT | FALSE | -0.08 | 0.41 | 0.02 | 0.04 | 0.01 |
| GSM7088836 | PF-A | PF | M | Recurrence | FALSE | TRUE | -0.45 | 11.7 | 1 | 6.72 | 0.24 | 0.34 | COLD | TRUE | -0.45 | 0.51 | 0.04 | 0.04 | 0.02 |
| GSM7088837 | PF-A | PF | M | Presentation | FALSE | FALSE | 0.01 | 73.15 | 1 | 28.2 | 0.26 | 0.74 | COLD | TRUE | 0.01 | 0.51 | 0.04 | 0.06 | -0.03 |
| GSM7088838 | PF-A | PF | M | Recurrence | FALSE | FALSE | 0.09 | 113.85 | 1 | 28.39 | 1.2 | 1.26 | COLD | TRUE | 0.09 | 0.39 | 0.01 | 0.05 | -0.01 |
| GSM7088839 | PF-A | PF | M | Recurrence | FALSE | TRUE | -0.23 | 43.63 | 1 | 12.11 | 0.36 | 1.06 | HOT | TRUE | -0.23 | 0.75 | 0.04 | 0.07 | 0.01 |
| GSM7088840 | PF-A | PF | M | Presentation | FALSE | FALSE | 0.68 | 35.8 | 1 | 17.8 | 0.18 | 0.59 | HOT | FALSE | -0.1 | 0.68 | 0.03 | 0.07 | 0 |
| GSM7088841 | PF-A | PF | M | Presentation | FALSE | TRUE | -0.06 | 15.8 | 1 | 7.82 | 0.22 | 0.59 | HOT | TRUE | -0.06 | 0.44 | 0.02 | 0.04 | 0.01 |
| GSM7088842 | PF-A | PF | M | Recurrence | FALSE | FALSE | 0.08 | 36.24 | 1 | 13.23 | 0.22 | 0.44 | COLD | TRUE | 0.08 | 0.44 | 0.02 | 0.04 | 0 |
| GSM7088843 | PF-A | PF | F | Presentation | FALSE | TRUE | -0.08 | 33.55 | 1 | 17.19 | 0.2 | 0.29 | HOT | TRUE | -0.08 | 0.48 | 0.03 | 0.04 | 0.01 |
| GSM7088844 | PF-A | PF | M | Presentation | FALSE | FALSE | 0.1 | 94.29 | 1 | 46.75 | 0.87 | 1.04 | HOT | TRUE | 0.1 | 0.33 | 0.01 | 0.05 | -0.03 |
| GSM7088845 | PF-A | PF | M | Presentation | FALSE | TRUE | -0.14 | 12.01 | 1 | 6.67 | 0.24 | 0.59 | COLD | TRUE | -0.14 | 0.66 | 0.03 | 0.06 | 0.02 |
| GSM7088846 | PF-A | PF | M | Presentation | FALSE | FALSE | 0.01 | 17.52 | 1 | 10.93 | 0.12 | 0.9 | COLD | TRUE | 0.01 | 0.55 | 0.03 | 0.05 | 0.01 |
| GSM7088847 | PF-A | PF | M | Presentation | FALSE | TRUE | -0.08 | 29.59 | 1 | 12.92 | 0.4 | 0.98 | COLD | TRUE | -0.08 | 0.27 | 0.01 | 0.03 | 0.01 |
| GSM7088848 | PF-A | PF | M | Presentation | FALSE | TRUE | -0.16 | 26.59 | 1 | 12.89 | 0.34 | 0.43 | COLD | TRUE | -0.16 | 0.48 | 0.04 | 0.04 | 0 |
| GSM7088850 | PF-A | PF | M | Recurrence | FALSE | TRUE | -0.08 | 59.12 | 1 | 18.02 | 1.05 | 0.31 | COLD | TRUE | -0.08 | 0.45 | 0.03 | 0.05 | -0.01 |
| GSM7088853 | PF-A | PF | M | Recurrence | FALSE | FALSE | 0.52 | 7.74 | 1 | 8.8 | 0.25 | 0.21 | COLD | FALSE | -0.18 | 0.52 | 0.04 | 0.04 | 0.01 |
| GSM7088859 | PF-A | PF | M | Presentation | FALSE | FALSE | 0.5 | 7.98 | 1 | 7.65 | 0.13 | 0.16 | HOT | FALSE | 0.02 | 0.5 | 0.03 | 0.05 | 0 |
| GSM7088861 | PF-A | PF | M | Recurrence | FALSE | FALSE | 0.29 | 34.31 | 1 | 19.61 | 0.23 | 0.57 | COLD | FALSE | 0.11 | 0.29 | 0.02 | 0.04 | -0.02 |
| GSM7088866 | PF-A | PF | M | Recurrence | FALSE | TRUE | -0.06 | 19.07 | 1 | 10.9 | 0.2 | 0.68 | COLD | TRUE | -0.06 | 0.65 | 0.04 | 0.06 | 0.01 |
| GSM7088870 | PF-A | PF | M | Presentation | FALSE | TRUE | -0.05 | 27.37 | 1 | 13.05 | 0.18 | 0.46 | COLD | TRUE | -0.05 | 0.4 | 0.02 | 0.04 | 0.01 |
| GSM7088871 | PF-A | PF | M | Recurrence | FALSE | TRUE | -0.09 | 17.15 | 1 | 11.29 | 0.23 | 1.2 | COLD | TRUE | -0.09 | 0.27 | 0.02 | 0.02 | 0.01 |
| GSM7088872 | PF-A | PF | F | Presentation | FALSE | TRUE | -0.04 | 12.56 | 1 | 10.47 | 0.31 | 0.62 | COLD | TRUE | -0.04 | 0.39 | 0.03 | 0.03 | 0.01 |
| GSM7088875 | PF-A | PF | F | Recurrence | FALSE | FALSE | 0.01 | 27.42 | 1 | 10.97 | 0.17 | 0.78 | HOT | TRUE | 0.01 | 0.43 | 0.02 | 0.04 | 0.01 |
| GSM7088876 | PF-A | PF | M | Presentation | FALSE | TRUE | -0.18 | 18.78 | 1 | 9.91 | 0.11 | 0.99 | HOT | TRUE | -0.18 | 0.6 | 0.03 | 0.05 | 0.01 |
| GSM7088879 | PF-A | PF | M | Presentation | FALSE | FALSE | 0.05 | 16.76 | 1 | 11.91 | 0.14 | 0.57 | HOT | TRUE | 0.05 | 0.39 | 0.02 | 0.04 | 0.01 |
| GSM7088880 | PF-A | PF | M | Presentation | FALSE | FALSE | 0.03 | 38.2 | 1 | 14.5 | 0.57 | 0.3 | HOT | TRUE | 0.03 | 0.4 | 0.02 | 0.04 | 0 |
| GSM7088884 | PF-A | PF | M | Presentation | FALSE | TRUE | -0.09 | 26.67 | 1 | 19.97 | 0.21 | 0.6 | HOT | TRUE | -0.09 | 0.69 | 0.03 | 0.06 | 0.01 |
| GSM7088886 | PF-A | PF | M | Presentation | FALSE | FALSE | 0.06 | 28.23 | 1 | 11.08 | 0.24 | 0.17 | HOT | TRUE | 0.06 | 0.46 | 0.01 | 0.05 | 0 |
| GSM7088887 | PF-A | PF | M | Presentation | FALSE | FALSE | 0.03 | 7 | 1 | 9.63 | 0.03 | 0.87 | HOT | TRUE | 0.03 | 0.3 | 0.02 | 0.02 | 0.01 |
| GSM7088888 | PF-A | PF | M | Presentation | FALSE | TRUE | -0.07 | 13.41 | 1 | 10.73 | 0.21 | 0.68 | HOT | TRUE | -0.07 | 0.58 | 0.02 | 0.06 | 0.01 |
